# RBM39 Functions as a Potential Oncogene Through the NF-κB Signaling Pathway in Colorectal Cancer Cells

**DOI:** 10.7150/jca.105120

**Published:** 2025-03-29

**Authors:** YaTao Wang, XueSi Yang, ZhangQuan Yang, ZiRui Chen, HaiFeng Jiang, YiCong Wang, DongYan Shen, GuoQiang Su

**Affiliations:** 1 Department of Colorectal Tumor Surgery, The First Affiliated Hospital of Xiamen University, School of Medicine, Xiamen University, Xiamen,361003, Fujian Province, China.; 2 Xiamen Cell Therapy Research Center, The First Affiliated Hospital of Xiamen University, School of Medicine, Xiamen University, Xiamen, 361003, Fujian Province, China.; 3 Department of Clinical Medicine, Fujian Medical University, Fuzhou, 350122, China.; 4 The Sixth People's Hospital of Luoyang, 471003, Luoyang, Henan Province, China.; 5 Department of Critical Care Medicine, Second People's Hospital of Yibin City, Yibin, 644000, Sichuan Province, China.; 6 Gastrointestinal Oncology, The Affiliated Hospital of Qinghai University, Xining 810000, Qinghai Province, China.

**Keywords:** RBM39, NF-κB, Proliferation, Migration, Invasion, Apoptosis, Colorectal cancer

## Abstract

Colorectal cancer (CRC) ranks as the third most frequently diagnosed cancer and is the second leading cause of cancer-related deaths globally. Recently, RNA-binding protein 39(RBM39), a critical factor in tumor-targeted mRNA and protein expression, has played a vital role in tumorigenesis and has broad development prospects in clinical treatment and drug research. However, the functional roles of RBM39 in the progression of CRC remain largely unexplored. This study found that RBM39 is notably overexpressed at both the mRNA and protein levels in CRC tissues compared with normal adjacent tissues. RBM39 was identified as a potential therapeutic target for colorectal cancer. Elevated RBM39 mRNA levels in CRC patients indicated worse survival probabilities. We show that RBM39 enhances the proliferation, migration, and invasion ability of CRC cells. Furthermore, we have made an innovative discovery that increased RBM39 inhibits apoptosis in CRC cells. Mechanistically, RNA-seq analysis indicated that RBM39 activates the NF-κB pathway, which plays a pivotal role in driving the malignant biological behaviors of colorectal cancer. Notably, these findings represent a novel contribution to our understanding of the mechanistic underpinnings of CRC, as they have not been previously documented in the literature. In the *in vivo* nude mouse xenograft model, our study demonstrates that the targeted knockdown of RBM39 markedly suppresses tumor formation, highlighting a novel therapeutic strategy for combating colorectal cancer. In conclusion, RBM39 emerges as a promising candidate for clinical diagnosis and targeted treatment of colorectal cancer, with implications for future research in tumor biology and therapeutic strategies.

## Introduction

CRC is a highly prevalent malignancy worldwide. In 2020, CRC (including anal cancer) accounted for over 1.9 million new cases and 935,000 deaths, constituting approximately 10% of all cancer diagnoses and deaths. It is the third most prevalent cancer globally and the second leading cause of cancer-related mortality 1. Currently, the treatment modalities for CRC primarily consist of surgical intervention, postoperative adjuvant therapy, and conversion therapy. Surgery continues to be the mainstay of treatment for CRC, while tumor recurrence and metastasis significantly impact patients' long-term survival 2, 3. The prognosis for advanced CRC remains poor, although screening strategies and CRC treatments have recently improved 4. Improved chemotherapy regimens have shown effectiveness for CRC patients who have lost the opportunity for surgery 5. However, it did not significantly improve their survival prognosis. Therefore, it is crucial to gain a deeper understanding of the molecular mechanisms underlying CRC initiation and progression for discovering novel, effective treatments and improving CRC patients' prognoses.

RNA binding motif protein 39 (RBM39), also referred to as HCC1 (hepatocellular carcinoma 1) and CAPER, was first identified as a novel nuclear antibody purified from liver cancer patients. It is a conserved RNA-binding protein involved in metabolism and the development of sensory neurons during embryogenesis 6, 7. Studies have shown that RBM39 is an RNA-binding protein widely involved in transcriptional co-regulation, selective RNA splicing, and protein translation 8, 9. Notably, reprogramming-induced changes in selective splicing and transcriptional expression levels are essential to cancer cell survival and development 10, 11. Therefore, splicing factors are actively being investigated as potential targets for anticancer therapies. RBM39 is rich in SR domains, including RNA recognition motifs (RRM) and one C-terminal U2AF homology motif 12. Studies have shown that aryl-sulphonamides, which is a class of organic compounds that contain an aryl group and a sulphonamide group, widely used in anticancer drug research, exert their anticancer effects by recruiting RBM39 to bind DCAF15 through structural complementarity with the α-helix of RBM39's RRM2 domain 13, 14. RBM39 is widely expressed and functions as an oncogene in multiple cancers, including breast cancer 15, liver cancer 16, acute myeloid leukaemia^17^, lung cancer^18^. RBM39, as an RNA-binding protein (RBP), interacts with RNA via its RNA-binding domains. It significantly promotes various critical cellular processes, such as development, differentiation, proliferation, cell cycle progression, apoptosis, and angiogenesis 19, 20. Apart from its splicing function, RBM39 was initially recognized as a coactivator of activator protein-1 (AP-1) and the estrogen receptor (ER). It has also been found to affect the transcriptional activity of steroid receptors and NF-κB^21^, ^22^. Moreover, RBM39 boosts the transcriptional activity of several key transcription factors, including progesterone receptor (PR), estrogen receptor-α (ERα) and estrogen receptor-β (ERβ) 7. Despite its overexpression in CRC 23, the role and mechanism of RBM39 in the pathogenesis of CRC are remain to be elucidated.

Our study aimed to determine the role of RBM39 in CRC. We first demonstrated that RBM39 is upregulated in CRC and is associated with worse postoperative survival in CRC patients. *In vitro* experiments revealed that knockdown of RBM39 inhibited cell proliferation, the abilities of migration and invasion. Furthermore, knockdown of RBM39 inhibits CRC cells apoptosis. Conversely, overexpression of RBM39 led to the opposite effects. Mechanistically, we demonstrated that RBM39 influences the malignant biological behavior of colorectal cancer through the NF-κB signaling pathway. In nude mice xenograft model, RBM39 knockdown significantly suppressed tumor formation. These findings suggest that RBM39 could serve as a potential therapeutic biomarker for CRC and provide insights into the mechanisms by which RBM39 promotes CRC progression. Our results may aid in the development of new strategies for targeted therapy in CRC.

## Materials and methods

### Bioinformatics analysis

RNA-seq data from The Cancer Genome Atlas (TCGA) were employed to assess RBM39 expression through the online tool (http://gepia.cancer-pku.cn) and another online tool (http ://www.sxdyc.com/). The Kaplan-Meier survival curve online tool was used to analyze the prognoses of CRC patients with different levels of RRBM39 mRNA expression.

### Cell culture and cell line

Normal human colon epithelial cells (NCM460), along with seven human CRC cell lines (SW620, SW116, Caco2, SW480, HCT116, RKO, HT29), and human embryonic kidney 293T cells, were sourced from the Cell Bank of the Chinese Academy of Sciences (China). These cell lines were cultured in DMEM (BasalMedia, China) with 10% fetal bovine serum (FBS, BasalMedia, China) and 1% antibiotics (100 IU/mL penicillin, 100 µg/mL streptomycin, BasalMedia, China). Cells were incubated at 37°C with 5% CO2.

### Patients and tumor specimens

CRC tissues and paired adjacent normal tissues were collected following surgery, Tissues were Frozen in liquid nitrogen and fixed in 10% neutral formaldehyde. All specimens were sourced from the Department of Colorectal Tumor Surgery at the First Affiliated Hospital of Xiamen University. None of the patients had undergone preoperative chemotherapy or radiotherapy. Informed consent was obtained from all participants, and the study received approval from the hospital's ethical review board. The clinical information of CRC patients is summarized in Table S1. The patience inclusion and exclusion criteria are as follows. Inclusion Criteria: Age between 30 and 80 years; Pathologically confirmed diagnosis of colorectal cancer, with surgical treatment; adenocarcinoma; Stages I-III; No prior radiotherapy, chemotherapy, or immunotherapy before surgery; No severe cardiovascular diseases, diabetes, pulmonary diseases, or other significant comorbidities; No history of other malignancies. Exclusion Criteria: Patients diagnosed with colorectal cancer who have received radiotherapy, chemotherapy, or immunotherapy; Patients with severe cardiovascular diseases, pulmonary diseases, diabetes, or other significant comorbidities; History of other malignancies.

### Immunohistochemistry (IHC)

Tumor tissues from CRC patients and nude mice and paired normal tissues were embedded in paraffin. The tissue sections were subjected to heating, deparaffinization, and rehydration, followed by antigen retrieval using citrate buffer. Endogenous Peroxidase Blocking Buffer (Beyotime, Shanghai, China) was used to inhibit the Endogenous peroxidase activity for 15 min at room temperature. Following three washes with PBS, the sections were incubated overnight at 4°C with a primary antibody. Subsequently, the sections were incubated for 1 h at 37°C with a secondary antibody (Sigma, China). After staining with DAB (Maxim Biotechnologies, Fuzhou, China), the sections were counterstained with Mayer's hematoxylin, dehydrated, and mounted. Images were captured using a microscope (Zeiss, Oberkochen, Germany). The primary antibodies used for IHC included: anti-RBM39(21339-1-AP, 1:50 dilution, proteintech), anti-PCNA (sc-25280, 1:100 dilution, Santa Cruz), anti-Ki67(sc-23900, 1:100 dilution, Santa Cruz).

### Western blotting

Colorectal cells and tissues from patients were lysed using RIPA buffer (Solarbio, China) supplemented with protease inhibitor cocktail (Roche Diagnostics, China) and phosphatase inhibitor cocktail (Roche Diagnostics, China). BCA kit (Thermo Fisher Scientific, China) was used to calculate Total protein concentration. Then, protein lysates were transferred to 0.22 µm PVDF membranes (Millipore, Bedford, US) using eBlot protein transfer system (GenScript). The PVDF membrane was blocked with 5% skim milk for 2 h, followed by incubation with the primary antibody overnight at 4°C. Afterward, it was incubated with secondary antibodies for 1 h at room temperature, and detection was performed using an enhanced chemiluminescence reagent. The following primary antibodies were used: anti-GAPDH (D16H11, #5714S, CST), anti-GAPDH (60004-1-Ig, Proteintech), anti-RBM39 (21339-1-AP, Proteintech), anti-BAX (60267-1-Ig, Proteintech), anti-BCL2 (sc-7382, Santa Cruz), anti-PARP (9532S, CST), and anti-P65 (66095-1-Ig, Proteintech), anti-phosphorylated P65 (AP0684, ABclonal).

### Quantitative real-time reverse-transcription PCR (qRT-PCR)

Total RNA from CRC cells and patients were extracted using the Total RNA Kit (TIANGEN, DP419, China). Reverse transcription to cDNA was carried out with the FastQuant RT Kit (with gDNase) (TIANGEN, KR106, China) following the manufacturer's instructions. cDNA synthesis for qRT-PCR was performed using the SuperReal PreMix Plus Kit (TIANGEN, FP205, China) on the ABI 7500 Fast Fluorescence Thermocycler. Relative mRNA expression levels for each gene were calculated using the 2^-△△CT^ method, with GAPDH as the reference. All experiments were repeated in triplicate. The primers used for detecting human transcripts are listed in Table 1.

### Colony formation assay

Cells (1x10^3^ cells per well) were incubated for 10-14 days in a six-well plat. Then, 4 % polyoxymethylene was used to fix colonies for 10 min at room temperature. The colonies were washed three times with PBS and stained with 0.5% crystal violet for 5 min. The number of colonies was counted using ImageJ.

### Cell survival and proliferation detection

Cells were plated in triplicate into 96-well culture plates at a density of 1x10^3^ cells per well. According to the manufacturer's instructions, the Cell Counting Kit-8 (CCK-8) kit (Promega, China) was performed at designated time points (1, 2, 3, and 4 days) to evaluate cell survival and proliferation. After 2 h, the absorbance of colorectal cancer cells at 450 nm was measured using a spectrophotometer.

### Cell migration and invasion assay

5x10^4^ Cells were seeded into the upper chamber of a Transwell insert (8-mm pore size, corning Inc) containing serum-free medium. A medium with 10% FBS was subsequently added to the lower chamber as a chemoattractant. In contrast to the migration experiment, the invasion assay used a cell number of 10x10^4^ and the upper chamber contained Matrigel. After incubation at 37˚C with 5% CO_ 2_ for 48 h, the medium in the upper chamber was removed, and the chamber was washed with calcium-free PBS. Cells were fixed with methanol for 30 min and stained with 0.1% crystal violet for 20 min. Unmigrated cells in the upper chamber were gently wiped away with a cotton swab. The stained cells were then photographed using a microscope, and the number of cells was counted using ImageJ.

### Wound healing assay for CRC cell migration

After cells had grown to 100% confluency in a 6-well plate, the cell layer was scraped with a 10 µl pipette tip, and the medium with 10% FBS was replaced with serum-free medium. Images of the cells were captured at 0 and 48h. The widths of the scratch widths at 0 and 48 h were measured and analyzed using Image J.

### Beyoclicktm EDU

EdU staining was conducted using the BeyoClick™ EdU Cell Proliferation Kit (C0085S, Beyotime, China). Cells were Cultivated in 6-well plates and proceeded with subsequent steps once the cell density reached 60%. Cells were fixed with 4% paraformaldehyde for 10 min, then washed with 3% BSA in PBS. Permeabilization was performed using 0.5% Triton-X 100 in PBS for 20 min, followed by another wash. Cells were then incubated for 30 min in the dark with a reaction cocktail for Alexa Fluor® 488 azide and EdU. After another PBS wash, cell nuclei were stained with Hoechst 33342 (1:1000 in PBS) for 10 min and then washed again. The pictures were capture using fluorescence microscope (Leica, Germany).

### Lentiviral transduction and transfection

The shRNA for RBM39 plasmids、control vector PLKO, and packaging plasmids psPAX2 and pMD2.G were bought from MiaoLing Plasmid Platform. (xiaolongbao, co, Ltd, China), The human shRNA sequences to inhibit RBM39 expression are listed as follows: RBM39 shRNA-1: 5'-GCGAAGTAGAGACAGAGAAAG-3'; RBM39 shRNA-2: 5'-AGACGATATTGATATTGAAGC-3'; RBM39 shRNA-3: 5'-AGTCGAGATCGAAGATTTAGA-3'. The pLVX-Puro-Flag-RBM39 plasmid (NM 184234.3) and control vector pLVX plasmid were bought from Public Protein/Plasmid Library (Geneppl, co, Ltd.).

The vectors were packaged into 293 cells, and recombinant lentiviruses were produced by transiently transfecting HEK293T cells with Lipofectamine®3000 Reagent (Thermo Fisher Scientific, USA), according to manufacturer's protocol. To establish stable RBM39 knockdown and overexpression cell lines, 5 µg /mL puromycin (Solarbio, China) were used to select transduced cells for 3 days.

### Flow cytometry analysis

Cells treated with CCCP (20 µM, MCE, China) for 24h were trypsinized, collected, and washed with PBS for apoptosis detection. Cells were stained with Annexin V-FITC and PI dye for 15 min following user guide of Annexin V-FITC Apoptosis Detection Kit (BD Biosciences, CA, USA), and then measured by flow cytometer (Agilent, USA).

### RNA-sequencing analysis

Knockdown of RBM39 and control cells were plated in 10cm culture dishes for 48 h. The total RNA was extracted using a Trizol reagent kit (Thermo Fisher, China) after the cells were washed three times with ice-cold PBS. The subsequent RNA sample processing and RNA sequencing analysis services were provided by APTBIO(China). Differentially expressed genes (DEGs) were analyzed using DESeq2 software across six samples, comparing HT29 shCtrl with shRBM39. Genes were filtered using a false discovery rate (FDR, adjusted p-value ≤ 0.05, fold change ≥ 1). The Kyoto Encyclopedia of Genes and Genomes (KEGG) was applied to explore the signaling pathway of the RBM39-related DEGs in the RBM3 KEGG Mapper. To explore RBM39-mediated molecular pathways in colorectal cancer, Gene Set Enrichment Analysis (GSEA) was conducted using Broad Institute GSEA software version 4.0.

### Animal experiments

Nude mice (BALB/c, 14-16g) were acquired from SLAC Laboratory Animal Co. Ltd. (Shanghai, China). All experimental procedures were conducted in accordance with the approved animal handling protocols of the Laboratory Animal Center of Xiamen University and received approval from the Animal Ethics Committee of Xiamen University [XMYY-2022KYSB003]. For the xenograft experiment, HT29-pLKO.1 and HT129-pLKO.1-sh (2 × 10^6^ cells), in serum‑free DMEM medium, were injected subcutaneously into the left and right flanks of 4-6-week-old male BALB/c nude mice. Nude mice were randomized into 2 groups: shCtrl group and RBM39 knockdown group. Tumor growth was tracked every two days by measuring its length and width. The tumor volume was calculated according to the formula V (mm^3^) = 1/2 ×Length × Width^2^. After 3 weeks, the nude mice were euthanized by cervical dislocation. Tumors were excised, photographed, and weighed immediately. Then, the tumors were fixed in formalin for immunohistochemical analysis.

### Statistical analysis

All statistical analyses were conducted using GraphPad Prism 9 (GraphPad Software Inc., La Jolla, CA, USA). Data are expressed as mean ± Standard Deviation (SD) unless stated otherwise. The differences between two groups were analyzed using Student's t-test, while one-way ANOVA was employed for comparisons among multiple groups. Analysis of survival curve of CRC patients using the Kaplan-Meier Plotter online tool. *P* < 0.05 was considered statistically significant.

## Results

### RBM39 is overexpressed in CRC tissues and correlates with a poor prognosis

**Figure 1:**
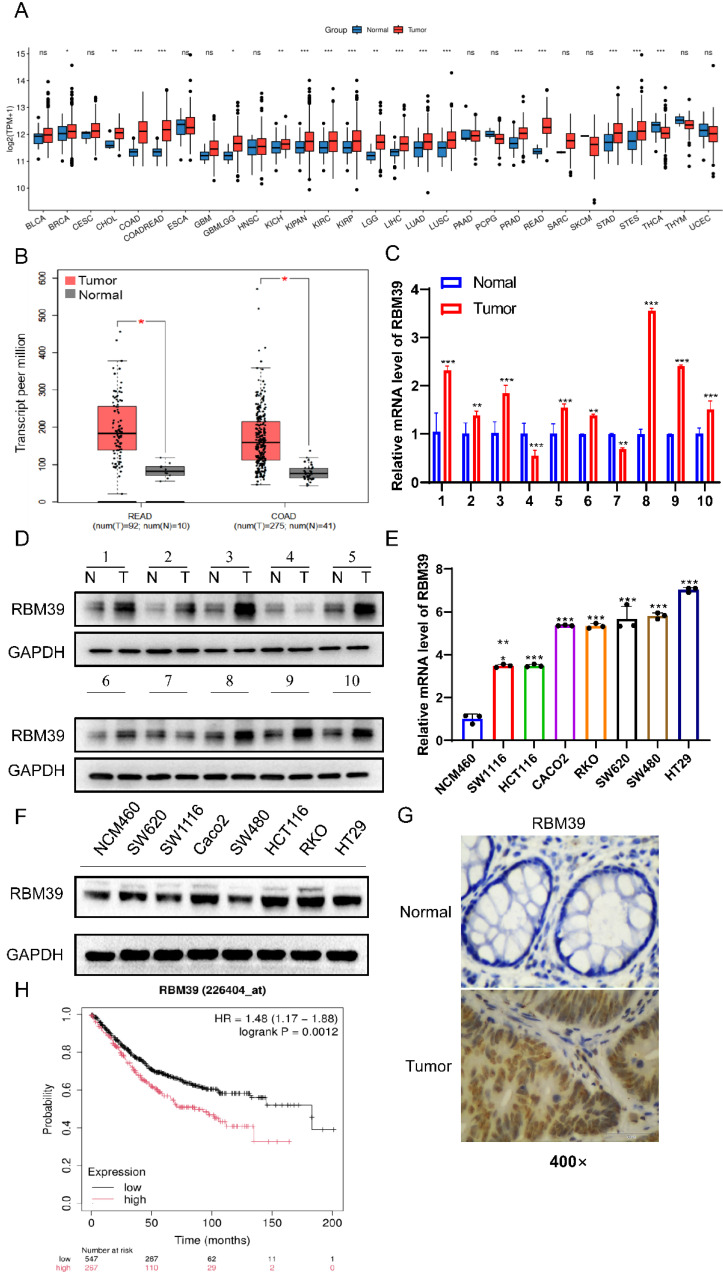
** RBM39 is overexpressed in CRC tissues and correlates with a poor prognosis.** (A) The online tool (http://www.sxdyc.com/) was used to analyze RBM39 mRNA expression across various tumors and normal tissues using TCGA datasets. (B) The GEPIA online tool was employed to analyze RBM39 mRNA expression in normal and CRC tissues using data from TCGA datasets. (C) The mRNA expression levels of RBM39 were measured by qRT-PCR in 10 pairs of colorectal cancer tissues and corresponding adjacent normal tissues (n = 3). (D) The protein expression levels of RRBM39 were measured by Western blot in 10 pairs of colorectal cancer tissues and corresponding adjacent normal tissues. (E) The mRNA levels of RBM39 were assessed in CRC cell lines (Caco2, RKO, SW620, SW1116, HT29, SW480, HCT116) and the normal colorectal cell line NCM460 using qRT-PCR (n = 3). (F) RBM39 protein levels were assessed in CRC cell lines (Caco2, RKO, SW620, SW1116, HT29, SW480, HCT116) and the normal colorectal cell line NCM460 using western blot. (G) Representative RBM39 is highly expressed in CRC tissues and compared with paired adjacent normal tissues using immunohistochemistry. Images under 400× magnification, scale bars, 50 µm. (H) The Kaplan-Meier online tool was utilized to perform survival analysis of CRC patients based on RBM39 mRNA expression. (N = adjacent normal tissue, T = tumor tissue, data are presented as the mean ± SD, the differences between two groups were analyzed using Student's t-test, *p < 0.05 **p <0.01, ***p <0.001).

To better understand the role of RBM39 in CRC, the expression level of RBM39 between normal and CRC tissues was detected. Analysis of The Cancer Genome Atlas (TCGA) database revealed that RBM39 mRNA expression levels were elevated in various tumor types (Fig. 1A), Moreover, the mRNA expression level of RBM39 was higher in CRC tissues compared with normal tissues (Fig. 1B). Meanwhile, we examined RBM39 expression levels in 10 pairs of CRC tissues and paired normal tissues. We found that RBM39 expression was elevated in 80% (8/10) of CRC tissues compared with normal tissues, both at the mRNA (Fig. 1C) and protein levels (Fig. 1D). Next, the protein and mRNA levels of RBM39 were assessed in human CRC cell lines (Caco2, RKO, SW620, SW1116, HT29, SW480, HCT116) and normal human colon epithelial cells (NCM460) using Western blot and qRT-PCR. Results indicated that RBM39 expression was significantly elevated in CRC cell lines, particularly in HT29 and RKO cells, at both the mRNA (Fig. 1E) and protein levels (Fig. 1F). Furthermore, IHC analysis revealed that RBM39 was overexpressed in CRC tissues compared with normal tissues. (Fig. 1G). Additionally, analysis using the Kaplan-Meier Plotter online tool indicated that patients with high RBM39 expression had a worse prognosis than those with low RBM39 expression. (Fig. 1H). Overall, RBM39 is overexpressed at both the mRNA and protein levels in CRC and acts as a significant prognostic marker for CRC patients.

In addition, we investigated whether RBM39 expression profiles could be linked to any specific clinical parameters in the enrolled patients (Table 2). We compared the baseline characteristics of patients with high versus low RBM39 expression and found no significant differences in pathological T stage (P = 0.676), pathological N stage (P = 0.355), or carcinoembryonic antigen (CEA) levels (P = 0.470). However, patients with high RBM39 expression had a significantly higher proportion of M1 disease (9.6% vs. 6.2%, P = 0.017) and a lower incidence of lymphatic invasion (17% vs. 22.9%, P = 0.009). In addition, the high-expression group showed a greater proportion of adenocarcinomas (47.1% vs. 39.8%, P < 0.001) and fewer mucinous adenocarcinomas (3.2% vs. 10%). Median age was significantly lower in the high-expression group (66.5 vs. 69 years, P = 0.031), whereas body mass index (BMI) did not differ significantly between the groups (P = 0.701). These findings suggest that RBM39 expression may be associated with specific clinicopathological features.

### RBM39 promotes the proliferation of CRC cells

**Figure 2:**
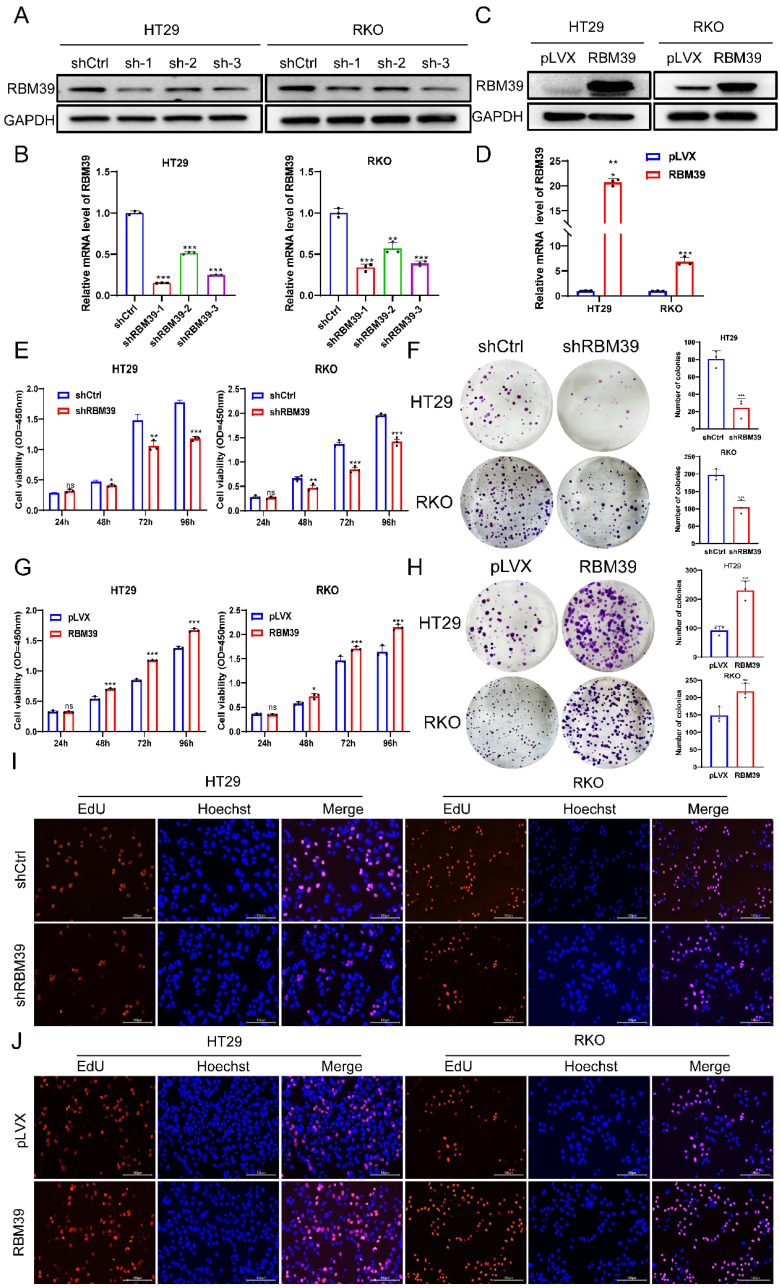

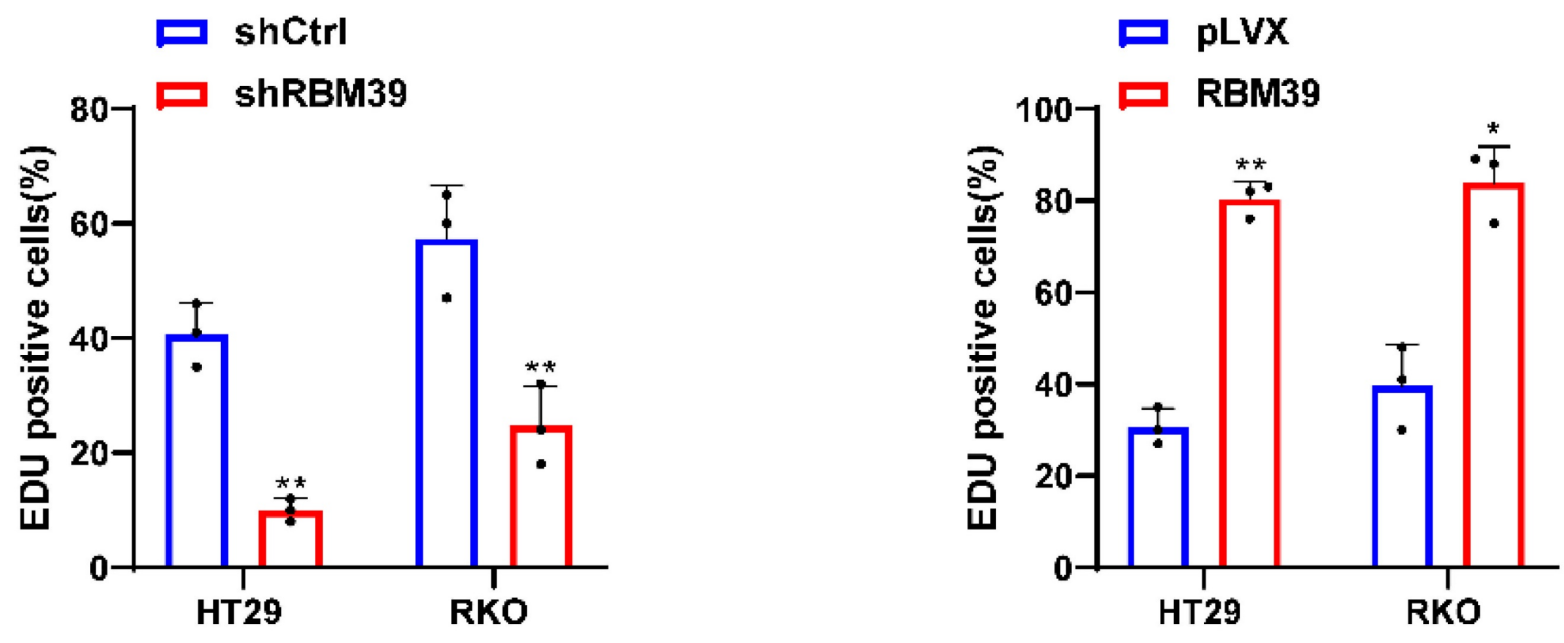
** RBM39 promotes the proliferation of CRC cells.** (A) The efficiency of RBM39 knockdown was confirmed at the protein level in HT29 and RKO cells using western blot. (B) The efficiency of RBM39 knockdown was confirmed at the mRNA level in HT29 and RKO cells using qRT-PCR (n = 3). (C) The efficiency of RBM39 overexpression was confirmed at the protein level in HT29 and RKO cells using western blotting. (D) The efficiency of RBM39 overexpression was confirmed at the mRNA level in HT29 and RKO cells using qPCR (n = 3). (E) The CCK8 assay demonstrated that RBM39 knockdown reduced the proliferation ability of HT29 and RKO cells compared with the control group, with measurements taken at 24, 48, 72, and 96 h (n = 3). (F) The Colony formation assay demonstrated that RBM39 knockdown reduced the proliferation ability of HT29 and RKO cells compared with the control group (n = 3), the number of colonies were counted. (G) The CCK8 assay demonstrated that RBM39 overexpression promoted the proliferation ability of HT29 and RKO cells compared with the control group, with measurements taken at 24, 48, 72, and 96 h (n = 3). (H) The Colony formation assay demonstrated that RBM39 overexpression promoted the proliferation ability of HT29 and RKO cells compared with the control group (n = 3). the number of colonies were counted (I) EdU assays were used to verify the proliferative ability after RBM39 was knocked down in HT29 and RKO cells at 100× magnification. Scale bars, 150µm (n = 3), the proliferating cells were counted. (J) EdU assays were used to verify the proliferative ability after RBM39 was overexpressed in HT29 and RKO cells at 100× magnification. Scale bars, 150µm (n = 3), the proliferating cells were counted. (Data are presented as the mean ± SD, the differences between two groups were analyzed using Student's t-test, *p < 0.05, **p < 0.01, ***p < 0.001).

Given the high RBM39 expression in CRC tissues and its impact on patient prognosis, we investigated the function of RBM39 in CRC cells. According to the mRNA and protein expression levels of RBM39 in CRC cells (Fig. 1E-F). We selected HT29 and RKO cells for knocking down experiments. RBM39 was efficiently knocked down by three shRNAs in HT29 and RKO CRC cells (Fig. 2A-B). shRBM39-1 was used for Subsequent experiments according to knockdown efficiency. Then, overexpression experiments in the base of HT29 and RKO were Conducted, and RBM39 was efficiently overexpressed. (Fig. 2C-D). The CCK-8 and colony formation assays showed that RBM39 knockdown inhibited the proliferation and clonogenic ability of HT29 and RKO cells compared with controls. (Fig. 2E-F). The converse holds true in the context of overexpression (Fig. 2G-H). Furthermore, the EdU staining also indicated that knockdown of RBM39 inhibits proliferation ability of HT29 and RKO Compared with control groups (Fig. 2I). In contrast, overexpression of RBM39 promotes the proliferation ability of HT29 and RKO Compared with control groups (Fig. 2J). These results indicate that RBM39 plays an important role in contributing to CRC cells proliferation.

### RBM39 promotes the migration and invasion of CRC cells

**Figure 3:**
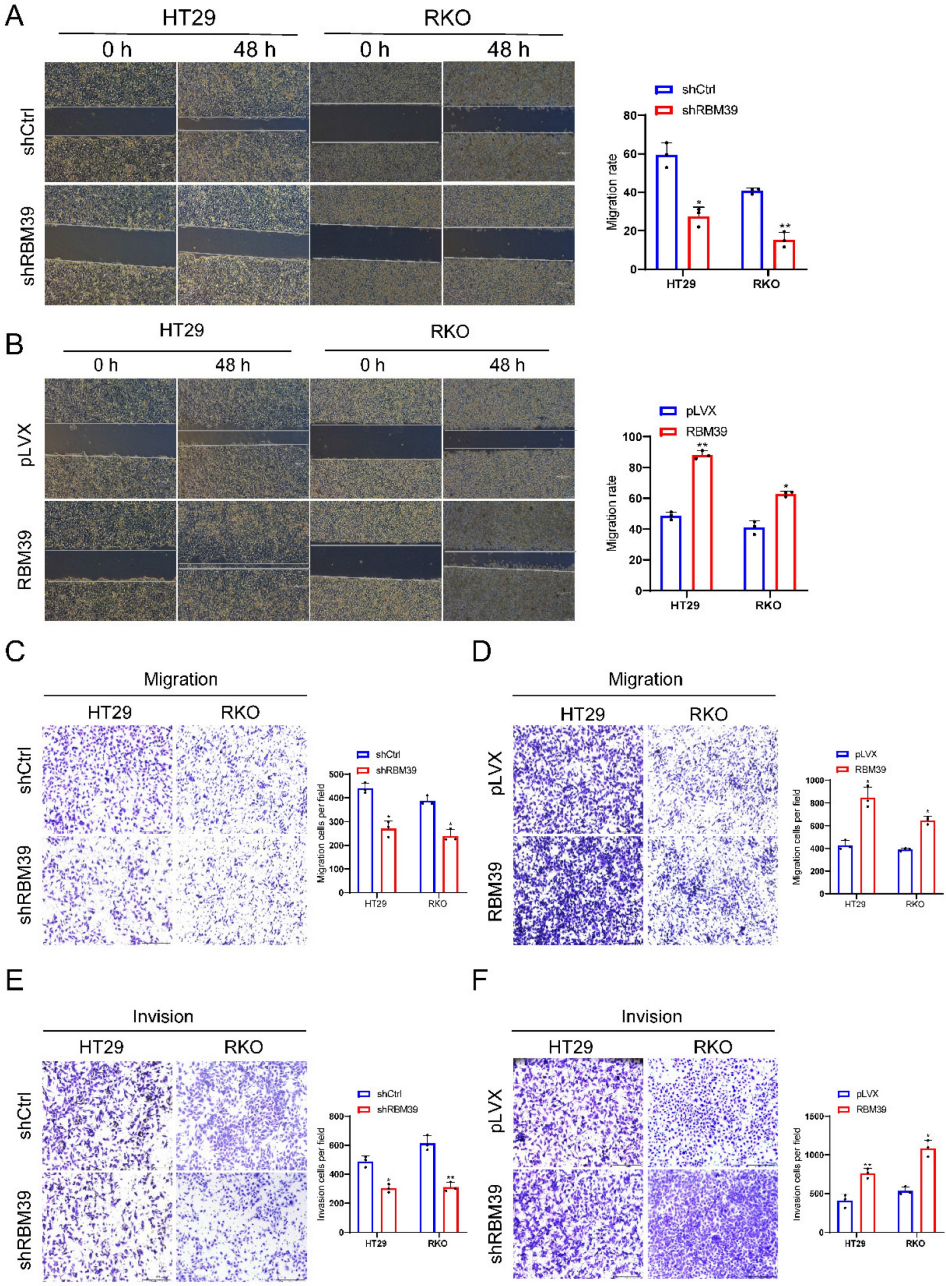
** RBM39 promotes the migration and invasion of CRC cells.** (A) Wound healing assay showed that RBM39 knockdown significantly decreased the migration ability of HT29 and RKO cells. Images were captured at 0 and 48 hours, and the scratch widths were analyzed. (n = 3). Scale bars, 150 µm. (B) Wound healing assay showed that RBM39 overexpression significantly promoted the migration ability of HT29 and RKO cells. Images were captured at 0 and 48 hours, and the scratch widths were analyzed. (n = 3). Scale bars, 150 µm. (C) Transwell assay showed that RBM39 knockdown significantly decreased the migration ability of HT29 and RKO cells by the transwell assay. The number of migrated cells was counted after 48 hours (n = 3). Scale bars represent 275 µm. (D) The Transwell assay showed that RBM39 overexpression significantly promoted the migration ability of HT29 and RKO cells by the transwell assay. Number of migrated cells were counted after 48h (n = 3). Scale bars, 275 µm. (E) RBM39 knockdown significantly decreased the invasion ability of HT29 and RKO cells by the transwell assay. Number of invasion cells was counted after 48h (n = 3). Scale bars, 275 µm. (F) RBM39 overexpression significantly promoted the V ability of HT29 and RKO cells by the transwell assay. Number of invasion cells was counted after 48h (n = 3). Scale bars, 275 µm. (Data are presented as the mean ± SD, the differences between two groups were analyzed using Student's t-test, *p < 0.05, **p < 0.01, ***p < 0.001).

To further investigate whether RBM39 influences the migration and invasion of CRC cells. Wound healing showed that the healing speed of RBM39 knockdown dramatically diminished compared with control groups in HT29 and RKO cells (Fig. 3A). In the context of overexpression, the converse holds true (Fig. 3B). Migration experiment also showed that RBM39 knockdown markedly reduced the number of migrated cells through the Transwell polycarbonate filter compared with control groups in HT29 and RKO cells (Fig. 3C). In the context of overexpression, the converse holds true (Fig. 3D). invasion experiment showed that the number of invaded cells penetrating the basement membrane was significantly decreased in RBM39 knockdown groups than control groups in the HT29 and RKO cells (Fig. 3E). The number of invaded cells penetrating the basement membrane was significantly higher when RBM39 is overexpressed (Fig. 3F). These results indicate that RBM39 facilitates the migration and invasion of CRC cells.

### RBM39 inhibits apoptosis of CRC cells

**Figure 4:**
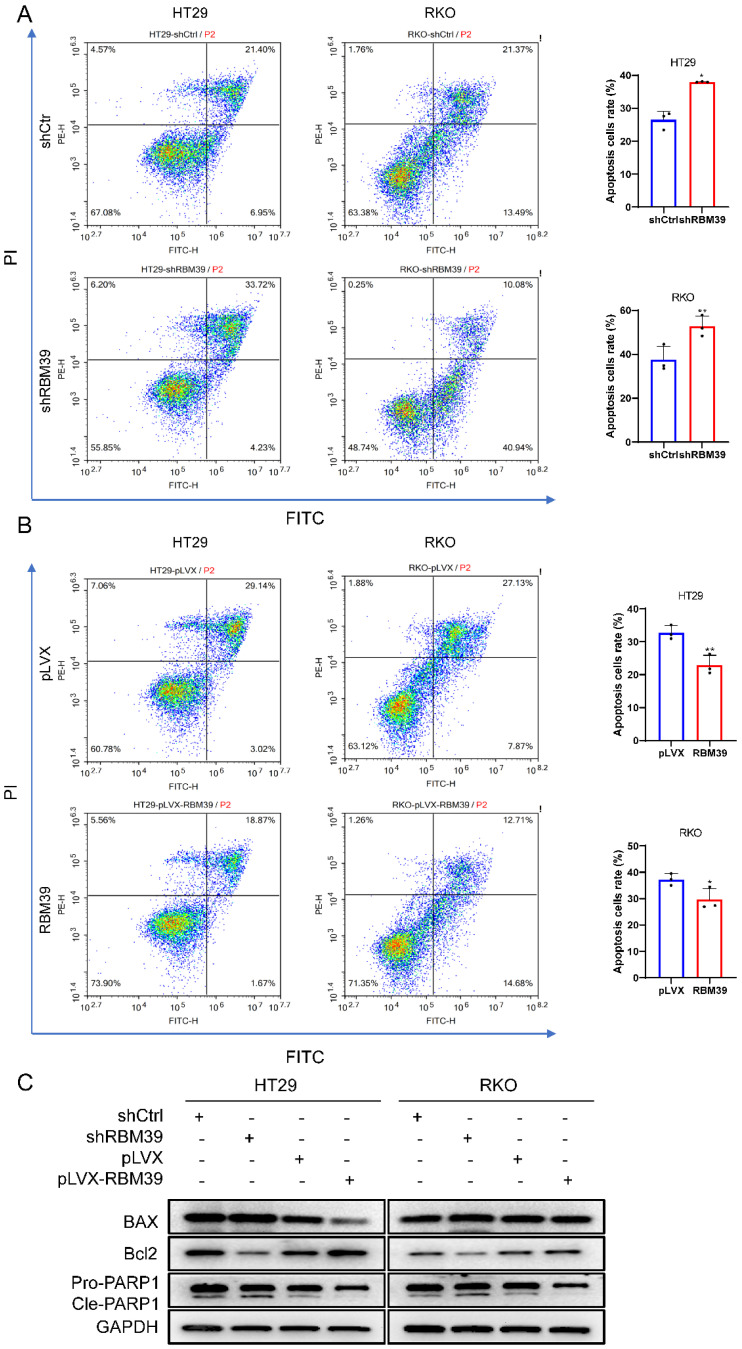
** RBM39 Inhibits apoptosis of CRC cells.** (A) The cell apoptosis was analysed after CCCP treatment for 48 h by flow cytometry analysis after RBM39 was knocked down in HT29 and RKO cells. (n = 3). (B) The cell apoptosis was analysed after CCCP treatment for 48 h by flow cytometry analysis after RBM39 was overexpressed in HT29 and RKO cells. (n = 3). (C) Western blot analysis of apoptotic proteins expression was performed in HT29 and RKO cells after CCCP treatment for 48 h. (Data are presented as the mean ± SD, the differences between two groups were analyzed using Student's t-test, *p < 0.05, **p < 0.01).

To further investigate whether RBM39 influences apoptosis of CRC cells. Apoptosis inducers CCCP was used to treat HT29 and RKO cells for 48 h. The apoptosis ratio detected by flow cytometry via Annexin-V-FTIC staining. Result showed that apoptosis level was significantly increased after RBM39 knockdown compared with control groups in HT29 and RKO cells (Fig. 4A). When RBM39 is overexpressed, its apoptosis level was significantly decreased (Fig. 4B). Furthermore, Western blot analysis also carried out to clarify the effects on apoptosis-related proteins when RBM39 is knocked down after treated with CCCP for 48h. We find that RBM39 knockdown increased protein expression levels of Bax, Cleaved PARP and decreased protein expression level of Bcl-2 in HT29 and RKO cells. The results are opposite when RBM39 is overexpressed (Fig. 4C). These results indicate that RBM39 mediates apoptosis resistance in CRC cells.

### RBM39 affects the progression of CRC through the NF-κB signaling pathway

**Figure 5:**
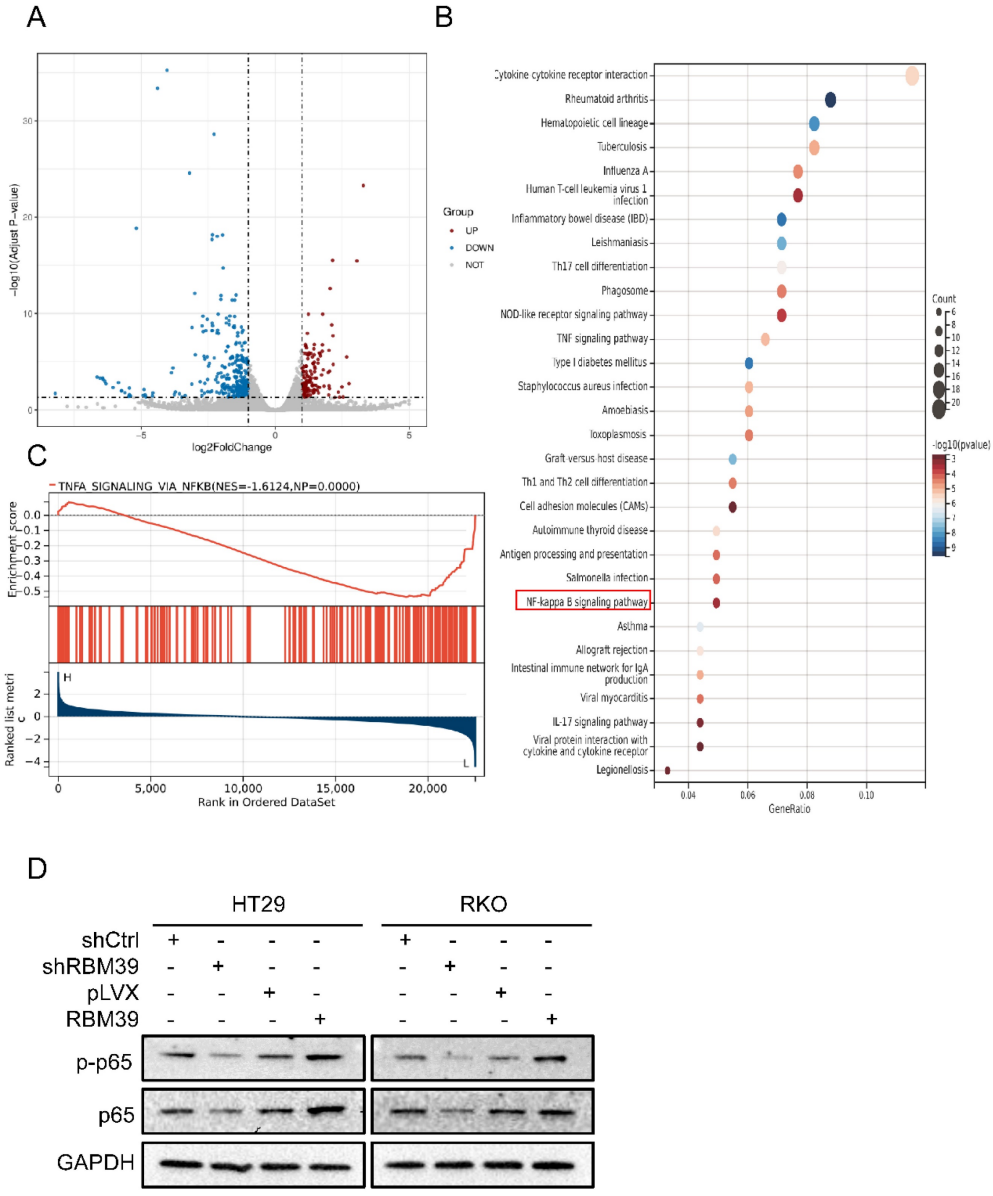
** RBM39 affects the progression of CRC through the NF-κB signaling pathway.** (A) RNA-seq analysis identified differentially expressed genes (DEGs) between HT29 shCtrl and shRBM39 cells, represented in a volcano plot. Genes with |log2 FC| > 1 and FDR < 0.05 were considered significant (Red: upregulated, log2FC ≥ 1; Blue: downregulated, log2FC ≤ -1; Grey: not significantly changed, -1 < log2FC < 1). (B) Identification of downstream signaling pathways of RBM39 was performed using KEGG analysis (C) GSEA was conducted to further investigate the connection between RBM39 and the NF-κB signaling pathway. NES (normalized enrichment score) and NP (normalized P-value) were used as indicators, with positive and negative NES values reflecting higher and lower expression levels, respectively. (D) Western blot was used to verify the expression levels of NF-κB signaling pathway-related proteins when RBM39 is knocked down or overexpressed.

To investigate how RBM39 affects CRC malignant biological progression, we conducted RNA sequencing on HT29-shCtrl and HT29-shRBM39 cells. RNA sequencing, filtered for FDR < 0.05 and |logFC| > 1, demonstrated that RBM39 knockdown notably impacted gene expression. We identified 325 genes that were upregulated and 136 genes that were downregulated. (Fig. 5A). In addition, KEGG pathway enrichment analysis revealed that the differentially expressed genes were significantly enriched in several pathways, including the TNF, NF-κB, and IL-17 signaling pathways (Fig. 5B). This indicates that RBM39 influences the progression of CRC through multiple pathways. Previous studies have shown that NF-κB plays a crucial role in tumorigenesis and tumor progression, which is closely associated with the occurrence and advancement of various cancers. 24. Therefore, we speculate that RBM39 affects the progression of CRC by influencing the NF-κB signaling pathway. Furthermore, To further confirm the relationship between RBM39 and NF-κB, GSEA analysis was performed on the HT29 shCtrl and shRBM39 groups (Fig. 5C). The results indicated that knockdown of RBM39 led to a decrease in the expression of NF-κB signaling pathway-related mRNAs. Subsequently, the impact of RBM39 on the NF-κB pathway was validated using Western blot. We find that the expression of NF-κB signaling pathway-related proteins P65 and p-p65 are decreased after RBM39 is knocked down, but the results are opposite when RBM39 is overexpressed (Fig. 5D). These results demonstrated that RBM39 promotes the malignant biological progression of CRC through the NF-κB signaling pathway.

### RBM39 knockdown suppresses tumor growth in subcutaneous xenograft tumor model

**Figure 6:**
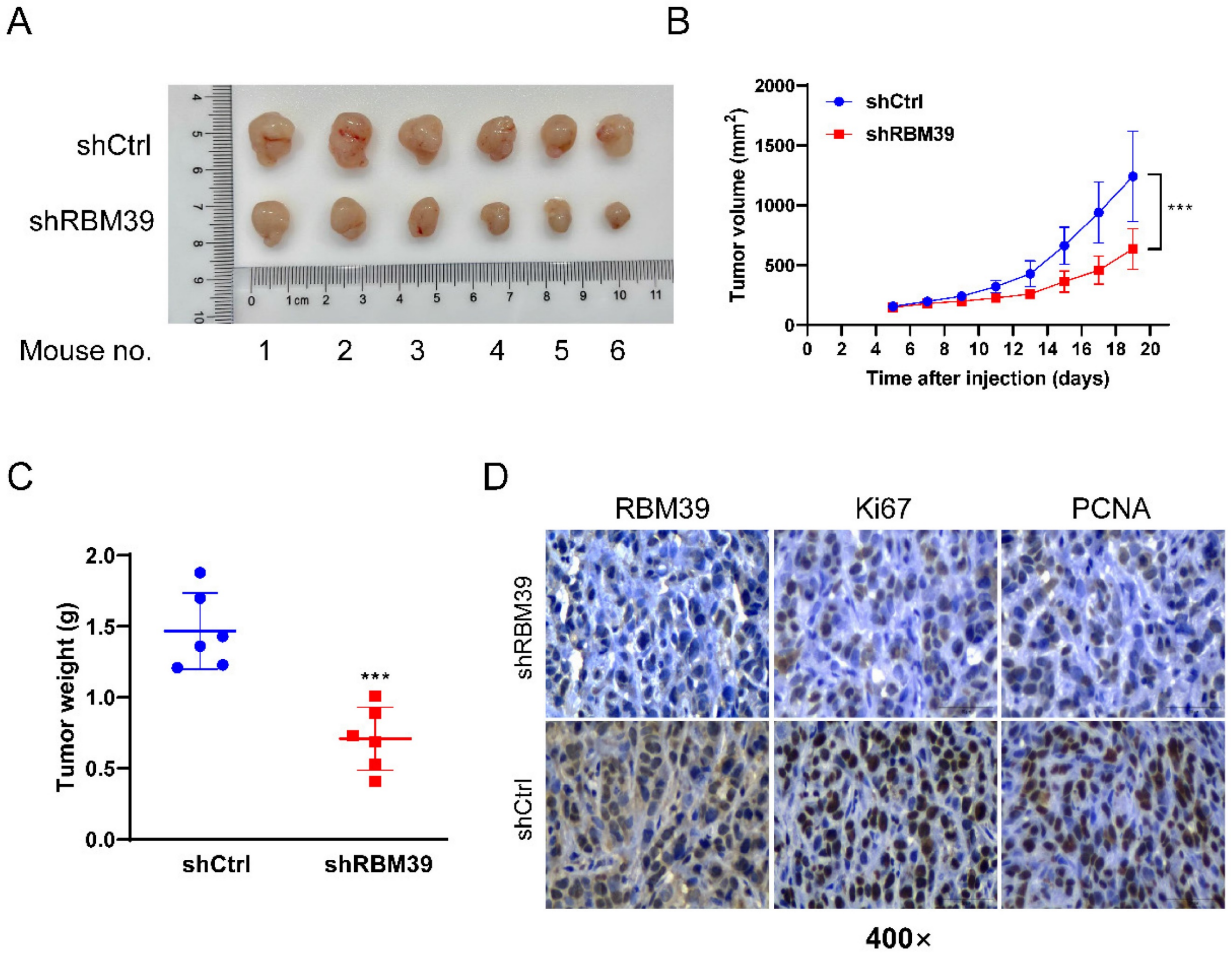
** RBM39 Knockdown suppresses tumor growth in subcutaneous xenograft tumor model.** (A) Photographs were taken of subcutaneous xenograft tumors in nude mice. (B) Tumor volume growth curves were measured every 2 days in nude mice xenograft models established with HT29-shRBM39 and control cells (n = 6). (C) Tumor weights were measured for statistical analysis at harvest time. (D) The efficiency of knocking down RBM39 and its effect on Ki67 and PCNA were confirmed by IHC staining at 400× magnification. Scale bars, 50µm. (Data are presented as the mean ± SD, the differences between two groups were analyzed using Student's t-test, ***p < 0.001).

To further validate the oncogenic role of RBM39 *in vivo*, HT29 cells with control or RBM39 knockdown were injected subcutaneously into the left and right flanks of nude mice. Respectively, Xenograft tumors derived from HT29 cells with RBM39 knockdown exhibited slower growth and smaller mean volumes and weights compared with tumors from control group (Fig. 6A-B). IHC staining verified the effectiveness of RBM39 knockdown and the proliferation status in xenograft tumors. (Fig. 6C). Our results revealed that RBM39 knockdown suppresses the proliferative capacity of subcutaneous xenograft tumors *in vivo*.

## Discussion

The prognosis of CRC patients is closely related to the stage of the tumor. For patients with stage I, the 5-year survival rate can reach 90%. But this rate decreases to 72% for patients with stage II-III and drops to 15% for those with stage IV 25. Despite continuous advancements in CRC treatment methods, significant challenges remain in the treatment of CRC. A substantial proportion of patients are diagnosed at an advanced stage, resulting in poor prognosis 26.

Therefore, further research is urgently needed to identify new targets and mechanisms for the treatment and diagnosis of CRC. RBM39 is an SR-rich RNA-binding protein that serves as both a pre-mRNA splicing factor and a transcriptional co-activator 16. Extensive research on RBM39 has highlighted its crucial roles in transcriptional regulation, alternative splicing, and protein translation 27. Moreover, RBM39 is upregulated in various tumors compared with normal tissues in various cancers 8. The abnormal overexpression of RBM39 in various tumors is closely associated with tumorigenesis, progression, and prognosis 9. In addition, recent studies have demonstrated that aryl sulphonamides, including indisulam, E7820, chloroquinoxaline sulfonamide (CQS), and tasisulam, target RBM39 to exert anti-tumor effects 28. Aryl sulphonamides function as molecular glue for the RBM39-DCAF15 E3 ubiquitin ligase complex, facilitating the targeted degradation of RBM39 and resulting in anti-tumor effects 28. Preclinical studies have demonstrated that aryl sulphonamides reduce RBM39 levels in cancer cells, leading to extensive splicing errors and a marked inhibition of cancer cell proliferation both *in vitro* and *in vivo* 29, highlighting a promising therapeutic strategy. RBM39 plays a critical role in the emerging field of molecular drug development 30, 31. In CRC cells HCT116, anticancer sulfonamides recruit DCAF15 to target and degrade RBM39, thereby inhibiting HCT116 proliferation 28. However, the potential phenotypes and mechanisms of RBM39 in CRC are little unknown, so it is necessary to investigate the role of RBM39 further in the development and progression of CRC. This research could positively impact the understanding of CRC etiology and treatment. Our study aims to address this gap.

In this study, we utilized the online tools for bioinformatics analysis and discovered elevated RBM39 expression in CRC. We also observed frequent overexpression of RBM39 in CRC cell lines and primary CRC tissues, aligning with previous findings. Studies showed that RBM39 is elevated in colorectal cancer and promotes the transition from adenoma to adenocarcinoma 23. Furthermore, the overexpression of RBM39 in CRC patients was identified as an independent factor predicting a poor prognosis. Subsequently, we investigated the role of RBM39 in CRC by examining its impact on cellular biological behavior. We demonstrated that knocking down RBM39 inhibits the proliferation of CRC cells and suppresses their migration and invasion *in vitro*. In contrast, overexpressing RBM39 produces the opposite effect. These findings are consistent with previous research. Wu *et al.* found that the anti-cancer sulphonamide E7820 reduced integrin α2β1 expression and suppressed metastasis of colorectal cells through mechanisms involving RBM39^32^. Chai *et al.* found that Overexpression of RBM39 in human NSCLC cell line H1299 significantly increased cancer cell proliferation and migration *in vitro* 33. Furthermore, xenograft study also demonstrated that knocking down RBM39 significantly inhibits tumorigenesis. These indicate that RBM39 acts as an oncogene promoting the occurrence and development of CRC.

Cell death, particularly apoptosis, is a key focus in cancer research. In cancer, there is a loss of balance between cell division and cell death. Cells that should undergo death do not receive the signals for apoptosis, leading to uncontrolled cell growth and, ultimately, tumor formation 34. In this experiment, we demonstrated that under the action of the apoptosis inducer Carbonyl cyanide 3-chlorophenylhydrazone (CCCP), knocking down RBM39 significantly promoted the Apoptosis ratio of CRC cells compared with control groups in HT29 and RKO through flow cytometry. Conversely, overexpression of RBM39 has the opposite effect. During the process of apoptosis, the balance between pro-apoptotic and anti-apoptotic protein regulators is crucial in determining whether a cell undergoes apoptosis. In tumor development, tumor cells typically evade programmed cell death by suppressing apoptosis through altering apoptosis signaling pathways, modulating the expression of apoptosis-related proteins, and interfering with cell death receptors, thereby escaping the fate of programmed cell death and continuing unrestricted proliferation 35. Therefore, Western blot was applied to demonstrate that RBM39 knockdown markedly decreased the expression level of Bcl-2, which its downregulation promotes tumor apoptosis and increased the expression levels of cleaved caspase-3、Bax and cleavage of poly (ADP-ribose) polymerase (PARP), which their overexpression promotes tumor apoptosis compared with control groups. These findings align with previous research showing that inhibiting RBM39 with peptides that strongly bind to c-Jun and block full-length RBM39 access increased DNA damage marker γ-H2AX levels and promoted apoptosis in breast cancer cells 36. So, it indicates that RBM39 plays an important role in apoptosis of CRC.

However, the exact mechanism by which RBM39 affects biological behavior in CRC remains unclear. We employed transcriptomics to investigate the potential mechanistic role of RBM39 in the CRC. KEGG enrichment analysis displays suggest that NF-κB may be a potential signaling pathway for RBM39. GSEA further confirmed that NF-κB is a potential signaling pathway of RBM39. Previous studies had shown that NF-κB plays a crucial role in tumor tumorigenesis and progression. Aberrant activation of the NF-κB pathway is closely associated with the occurrence and advancement of various cancers. In some cases, excessive activation of NF-κB can promote tumor cell proliferation, survival, and metastasis while inhibiting apoptosis in tumor cells, thereby facilitating tumor development. Additionally, NF-κB can also regulate processes such as inflammation, immune responses, and angiogenesis, further influencing the tumor microenvironment and providing favorable conditions for tumor progression 37. An increasing number of research highlights the significant role of NF-κB in the occurrence and development of CRC, involving apoptosis, angiogenesis, metastasis, and proliferation, among other CRC-related processes 24, 38. The NF-κB pathway is one of the most well-defined signaling pathways, consisting of the classical and non-classical pathways. In the classical pathway, phosphorylation of p65 and nuclear translocation are considered essential for NF-κB activation and subsequent biological functions 39. Known phosphorylation modification sites of NF-κB p65 are diverse, and phosphorylation at different sites mediates the regulation of transcription of different genes by nuclear NF-κB, thereby impacting cellular biological functions 39. Subsequently, Western blot analysis was conducted to verify the expression levels of p-p65 and p65. The results indicated that RBM39 knockdown reduced the expression of both p-p65 and p65, whereas RBM39 overexpression had the opposite effect. Zhu *et al.* reported that MyD88 regulates CRC cell proliferation, migration, and invasion through the NF-κB/AP-1 signaling pathway 40. Ding *et al.* reported that cyclooxygenase-1 downregulation stimulates mitochondrial apoptosis via the NF-κB signaling pathway in CRC cells 41. These results suggest that RBM39 influences the occurrence and progression of CRC through the NF-κB signaling pathway. However, the study has limitations. In the KEGG enrichment analysis of sequencing results, pathways affected by RBM39 are notably related to immunity, including the IL-17 and TNF signaling pathways, indicating a need for further study. Lu *et al.* found that RBM39, a splicing factor, is linked to the infiltration of immune cells, with its expression negatively correlating with immune cell presence in the tumor microenvironment. This may reduce CD4+ T cell infiltration and impair their immune response. Additionally, RBM39 expression is associated with immune checkpoint gene expression, potentially affecting CD4+ T cell activation and function 42. Zhang *et al.* reported that the expression of RBM39 correlates with immune checkpoint genes across different cancer types, influencing tumor cells' ability to evade immune detection. Furthermore, RBM39 can modulate immune escape mechanisms by affecting the types and counts of infiltrating immune cells 43. So further research is needed to explore the relationship between RBM39 and immune cells in the context of CRC development. Moreover, to further explore NF-κB signaling pathway mechanism, rescue experiments are required. NF-κB signaling pathway inhibitors should be utilized to validate their effects on cell proliferation, migration, invasion, and apoptosis.

In summary, RBM39 is a potential therapeutic target for CRC. We have confirmed the frequent overexpression of the RBM39 protein and its prognostic value in CRC patients. Our findings suggest that RBM39 overexpression affects the proliferation, invasion, migration, and apoptosis of CRC cells by activating NF-κB pathway activity. This study elucidates a novel mechanism by which RBM39 regulates apoptosis in colorectal cancer cells, augmenting existing theories regarding its oncogenic role. It provides critical insights into the complex regulatory network of RBM39 in tumor biology. Based on our findings on the prognostic value of RBM39 and its impact on the occurrence and progression of colorectal cancer, there is potential for developing targeted detection methods that can assist clinicians in accurately assessing disease progression risk in colorectal cancer patients, thus facilitating personalized treatment strategies. Moreover, the research offers an experimental platform for the development of small molecule inhibitors of RBM39, such as aryl sulfonamides like indisulam, or RNA interference drugs, paving the way for the discovery of novel anti-tumor medications targeting RBM39. The findings of this study are likely to attract the attention of researchers across various fields, including bioinformatics, pharmacology, and clinical medicine, fostering interdisciplinary collaboration to explore the comprehensive applications of RBM39 in integrated cancer therapy. While larger studies are needed to validate these findings before clinical application.

## Supplementary Material

Supplementary table.

## Figures and Tables

**Table 1:** Primers designed for gene expression detection.

Gene	Species	Forward primer (5'-3')	Reverse primer (5'-3')
GAPDH	human	AGCGAAGTAGAAGCAAAGAGAG	CCTCGGATGCACTGTTAAA
RBM39	human	CCCTTCATTGACCTCAACTACA	ATGACAAGCTTCCCGTTCTC

**Table 2:** Correlation between RBM39 expression and clinical characteristics in TCGA-CRC patients

Characteristics	Low expression of RBM39	High expression of RBM39	P value
n	322	322	
Pathologic T stage, n (%)			0.676
T1&T2	67 (10.5%)	64 (10%)	
T3	213 (33.2%)	223 (34.8%)	
T4	40 (6.2%)	34 (5.3%)	
Pathologic N stage, n (%)			0.355
N0	191 (29.8%)	177 (27.7%)	
N1	69 (10.8%)	84 (13.1%)	
N2	61 (9.5%)	58 (9.1%)	
Pathologic M stage, n (%)			0.017
M0	252 (44.7%)	223 (39.5%)	
M1	35 (6.2%)	54 (9.6%)	
CEA level, n (%)			0.470
<= 5	135 (32.5%)	126 (30.4%)	
> 5	74 (17.8%)	80 (19.3%)	
Primary therapy outcome, n (%)			0.437
PD&SD	17 (5.4%)	21 (6.7%)	
PR&CR	141 (45.2%)	133 (42.6%)	
Histological type, n (%)			< 0.001
Adenocarcinoma	252 (39.8%)	298 (47.1%)	
Mucinous adenocarcinoma	63 (10%)	20 (3.2%)	
Perineural invasion, n (%)			0.445
No	71 (30.2%)	104 (44.3%)	
Yes	21 (8.9%)	39 (16.6%)	
Lymphatic invasion, n (%)			0.009
No	162 (27.8%)	188 (32.3%)	
Yes	133 (22.9%)	99 (17%)	
Age, median (IQR)	69 (60, 77)	66.5 (57, 75)	0.031
BMI, median (IQR)	27.014 (23.91, 31.045)	27.102 (23.907, 32.216)	0.701

## Abbreviations

CRC: Colorectal cancer
RBM39: RNA-binding protein 39
NF-κB: Nuclear Factor kappa B
HCC1: hepatocellular carcinoma 1
CAPER: Coactivator of Apparent Molecular Weight 62 kDa
SR: Serine/Arginine-rich protein
RRM: RNA recognition motifs
U2AF: U2 Small Nuclear RNA Auxillary Factor
DCAF15: DDB1 And CUL4 Associated Factor 15
RBP: RNA-binding protein
AP-1: coactivator of activator protein-1
ER: estrogen receptor
PR: progesterone receptor
Erα: estrogen receptor-α
Erβ: estrogen receptor-β
TCGA: The Cancer Genome Atlas
IHC: Immunohistochemistry
EdU: 5-Ethynyl-2'-deoxyuridine
CCCP: Carbonyl cyanide m-chlorophenyl hydrazone
PI: Propidium Iodide
KEGG: The Kyoto Encyclopedia of Genes and Genomes
DEGs: Differentially Expressed Genes
GSEA: Gene Set Enrichment Analysis
SD: Standard Deviation
CQS: chloroquinoxaline sulfonamide
α2β1: Alpha-2 Beta-1 receptor
NSCLC: Non-Small Cell Lung Cancer
γ-H2AX: Phosphorylated Histone H2AX
